# Manual validation finds ultra-long-read sequencing best enables faithful, population-level structural variant calling in *Drosophila melanogaster* euchromatin with nanopore

**DOI:** 10.1093/g3journal/jkag043

**Published:** 2026-03-10

**Authors:** James A Hemker, Hannah R Gellert, Jessica A Smiley-Rhodes, Bernard Y Kim, Dmitri A Petrov

**Affiliations:** Department of Developmental Biology, Stanford University, 450 Jane Stanford Way, Stanford, CA 94305, United States; Department of Biology, Stanford University, 450 Jane Stanford Way, Stanford, CA 94305, United States; Department of Genetics, Stanford University, 450 Jane Stanford Way, Stanford, CA 94305, United States; Department of Ecology and Evolutionary Biology, Princeton University, 1 Nassau Hall, Princeton, NJ 08544, United States; Department of Biology, Stanford University, 450 Jane Stanford Way, Stanford, CA 94305, United States; Chan Zuckerberg BioHub, 499 Illinois St, San Francisco, CA 94158, United States

**Keywords:** structural variation, population genomics, long-read sequencing, ultra-long reads, nanopore sequencing, *Drosophila melanogaster*

## Abstract

The increasing accessibility of long-read sequencing and the rapid development of automated variant callers are promoting the generation of population-level structural variation data. However, the effect of the length of long-reads on automated variant callers is not well understood, especially for non-human species. Here we show that only ultra-long long-reads, with read N50s greater than 50 kb, are capable of accurately calling structural variants of any size in *Drosophila melanogaster* euchromatin. We used Oxford Nanopore Technologies to long-read sequence eight, inbred *D. melanogaster* strains to extremely high coverage (mean 238×), and we then downsampled the reads to create read pools of different length distributions. We assembled genomes from these different read-length pools and used both read-based and assembly-based structural variant callers to call variants in each strain before merging the calls into population-level datasets. We manually validated over 2,300 putative structural variants to assess the precision of the variant calls across the different read-length distributions and to determine the cause and rates of false positive errors. We found that more than half of all structural-variant-calling errors stem from misaligned reads that contain mobile elements or are located in repetitive and complex regions. Overall, our results show that long reads should be at least three times longer than the largest transposable elements found in the genome in order to accurately call structural variants at the population level.

## Introduction

Structural variants are genomic rearrangements such as insertions, deletions, duplications, and inversions that are often defined as greater than 50 bp (Mahmoud et al. 2019; Ahsan et al. 2023). Historically, single-nucleotide polymorphisms (SNPs) have been the focus of genomic diversity metrics and studies, but further research has shown that structural variants affect a larger proportion of the genome than SNPs (Durbin et al. 2010; Pang et al. 2010; Catanach et al. 2019). Recent population genetics studies suggest that structural variants face stronger negative selection than SNPs (Chakraborty et al. 2019; Hämälä et al. 2021), and structural variation underpins many severe human diseases. Repeat expansions are implicated in both Parkinson’s disease (Alvarez Jerez et al. 2024) and Huntington’s disease (Walker 2007), and structural variants are the leading class of driver mutations in cancer, as they can fuse or disrupt oncogenes and repurpose regulatory elements for dysregulated gene expression (Cosenza et al. 2022). At the same time, structural variants have played critical roles as large-effect adaptive mutations across the tree of life (Kapun and Flatt 2019; Wellenreuther et al. 2019; Gorkovskiy and Verstrepen 2021; Hämälä et al. 2021; Shi et al. 2023; Dodge et al. 2024; Ferguson et al. 2024). A tissue-specific-enhancer deletion in freshwater sticklebacks led to the loss of pelvic spine armor (Chan et al. 2010). In the hominoid lineage, a transposable element insertion into the intron of a key tail development gene led to an alternative splicing isoform that played a significant role in the loss of tails in humans and apes (Xia et al. 2024). Within insects, a transposable element insertion was responsible for the melanization of British peppered moths (Hof et al. 2016). In *Drosophila*, chromosome-scale cosmopolitan inversions vary in frequency across latitudinal and altitudinal clines, and they have been linked to both thermal adaptation and seasonal adaptation (Kapun and Flatt 2019; Kapun et al. 2023; Nunez et al. 2024). Chimeric genes, which are new genes that form when distinct gene sequences are combined, have a long history of study in *Drosophila* (Long and Langley 1993; Wang et al. 2002; Arguello et al. 2006).

Despite their outsized role in genomic variation, structural variants have been difficult to study. Their size and location in often-repetitive regions of the genome has made them hard to map and detect using traditional short-read sequencing data. The evolution of long-read sequencing technologies over the last decade has greatly improved our ability to find and analyze structural variants. Long reads are able to capture large structural variants and the flanking genomic regions in single reads, making them detectable and alignable. The addition of extremely long reads has been a key innovation for gapless genome assemblies (Nurk et al. 2022; Koren et al. 2024). Furthermore, continuous improvements to sequencing chemistries have increased the accuracy of long-reads to the point where very small variants can be confidently detected (Kolmogorov et al. 2023).

Structural variants are primarily detected through aligning sequencing reads or whole genome assemblies against a reference assembly (Ahsan et al. 2023). Computational methods then identify structural variants by unique mapping signatures within these alignments. Most structural variant calling tools use alignments of long reads to a reference genome. However within the last few years, the process of de novo genome assembly has become more computationally tractable. Furthermore, this new era of haplotype-resolved (or even telomere-to-telomere) assemblies provides a new, highly accurate data type from which structural variants can be called (Ebert et al. 2021). Using these high-quality assemblies, assembly-based methods can detect much larger variants (such as major insertions or inversions) more easily than read-based methods (Chakraborty et al. 2018, 2019).

Within the last few years, the technical and financial costs of long-read sequencing have decreased such that population-level long-read datasets are feasible to generate for individual labs (De Coster et al. 2021). These datasets are essential for uncovering structural variantion polymorphisms, which better capture the dynamic evolutionary processes that shape structural variant diversity. When generating variant data at the population level, accuracy is paramount to avoid compounding effects of false calls when combining data across many individuals (Ahsan et al. 2023). Though SNP joint genotyping is a well-established procedure (Poplin et al. 2018), structural variants can be hard to compare across individuals and require more complex validation strategies (Jeffares et al. 2017; Kirsche et al. 2023; Zheng et al. 2024). While benchmarked structural variant sets have been established for humans (Collins et al. 2020; Zook et al. 2020; Olson et al. 2023), highly validated structural variant callsets do not exist for most other species. Furthermore, many structural variant-calling methods are benchmarked on human data, leaving uncertainty in their precision and accuracy for non-human species. Validation of individual, computationally derived variant calls can be achieved by manually inspecting reads or genome assemblies and their alignments (Belyeu et al. 2018, 2021). While manual validation and curation can identify structural variants with extremely high precision, it requires significant effort for genome-scale studies across many individuals (Bertolotti et al. 2020).

A primary determinant of variant-calling quality is read length, as exemplified by the structural variation boom that has occurred since moving from short-read sequencing to long-read sequencing. Of the two primary long-read technologies, Oxford Nanopore Technologies (ONT) long-read sequencing can have a much wider read-length distribution than PacBio’s high-fidelity (HiFi) sequencing. While HiFi reads have a maximum read length of 25 kb, ONT long reads have no hard limit, and reads larger than 1 Mb have been sequenced (Payne et al. 2019). Individual nanopore long-reads within the same sequencing run can range 1,000× from hundreds of bases to megabases in length. ONT long-read length is a function of many parameters, and the read-length distribution of a single sequencing run can be influenced by input sample quality, DNA extraction method, and library prep kit (Wang et al. 2021). Given the many factors that can impact long-read length, it is surprising that many structural variant studies do not provide any specific justifications for their long-read lengths beyond technical limitations (Long et al. 2018; Hämälä et al. 2021; Li et al. 2023; Mérot et al. 2023; Shi et al. 2023; Liang et al. 2024). While it is generally accepted that longer read lengths and higher quality assemblies will provide more accurate structural variant calls (Chakraborty et al. 2016; Jiang et al. 2021; Ahsan et al. 2023; Mahmoud et al. 2024), the read-length threshold that is necessary for accurate calls is not well known, especially in non-human species. As ONT read-length distributions are not all equivalent, understanding this threshold is essential for efficient and accurate studies of structural variants.

In this study, we used the model system of *Drosophila melanogaster* inbred lines to test the impact of different read lengths on the precision of automated structural variant calling at the population level. *D. melanogaster* has a rich suite of literature describing functionally adaptive structural variants, including the cosmopolitan inversions (Kennington et al. 2007; Kapun et al. 2016; Kapun and Flatt 2019) and transposable elements (Aminetzach et al. 2005; González et al. 2008, 2010; Schmidt et al. 2010; Casacuberta and González 2013). Transposable elements can vary in length and population frequency, and can be highly similar across copies in the genome (Mérel et al. 2020). Due to these characteristics, transposable element insertions and deletions are hard to align with sequencing data and are challenging to call accurately. Still, transposable elements are estimated to account for a significant proportion of structural variation in *D. melanogaster* (Chakraborty et al. 2018, 2019; Rech et al. 2022), and thus correctly calling them is a crucial task.

We additionally reasoned that calling structural variants in the compact and gene-dense euchromatic regions of the *D. melanogaster* genome would be an easier task than in larger vertebrate genomes which feature greater proportions of intronic and intergenic sequences. ONT long-read sequencing protocols have been developed for *D. melanogaster* to cheaply and efficiently sequence very long (>100 kb) reads (Kim et al. 2021). *D. melanogaster* has a small genome (estimated female size: ∼175 Mb Hoskins et al. 2002) that makes it financially and computationally tractable for deep long-read sequencing and genome assembly of multiple individuals. Finally, inbred lines are treated as effectively homozygous (i.e. haploid), simplifying many processes from genome assembly to read alignment to variant calling.

**Fig. 1.**
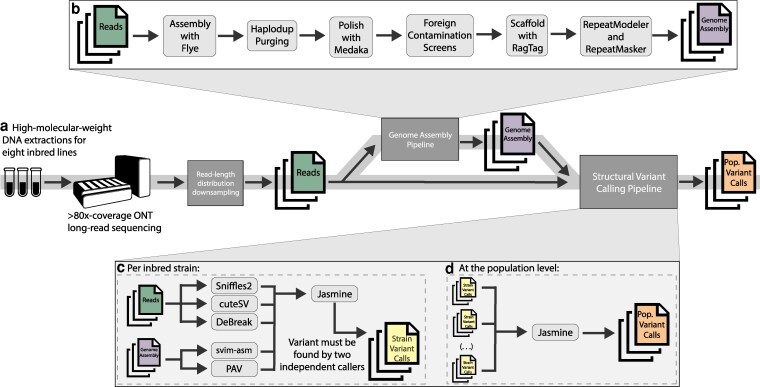
Overview of the methodological workflow. a) Each strain was sequenced to extremely high coverage with ONT long-read sequencing. Various read-length distribution read pools were made by computational downsampling. b) Reads were additionally assembled into genomes. c) Structural variants were called from both read and assembly alignments for each strain and each read-length distribution using five different callers. A variant had to be called by two different callers to be included in the final strain variant set. d) Variant calls from all strains were then merged into a final population variant set. Random variants from the final population set were manually validated.

Here, we sequenced eight *D. melanogaster* inbred strains to extremely high coverage with ONT using protocols to maximize read length. We then downsampled these deep read pools to generate 30×-coverage read pools with distinct read-length distributions, from which we additionally assembled genomes. We employed five, well-known, automated structural variant callers on each of these distinct read sets and genome assemblies (Fig. 1). By manually validating more than 2,300 putative structural variant calls and 16,000 genomic loci across the eight inbred lines, we determined the overall variant-calling precision for each read-length distribution. We found that structural variants called by the longest reads and their resulting assemblies were the most accurate, regardless of variant size. For variants smaller (<10 kb) than the majority of our long reads, we found that the rest of our read-length distributions were reasonably accurate. Larger variants (≥10 kb) could only be accurately called with the ultra-long data. We found that many of the false positives stemmed from misalignments of mobile elements or misalignments in highly complex, repetitive regions (Chakraborty et al. 2019).

## Methods

### DNA extraction and long-read sequencing

We sequenced *D. melanogaster* isofemale, inbred lines originally collected from Linvilla Orchard, PA, in 2011 (Behrman et al. 2015) (Supplementary Table 1). Flies for this study were reared at 25C on cornmeal-molasses fly media with a 12-hour day-night light cycle. Female flies were sexed and starved one day prior to DNA extraction.

We followed previously published *D. melanogaster* long-read sequencing protocols from our lab (Kim et al. 2021, 2024). Briefly, 100–400 female flies were homogenized using Kimble Kontes Dounce homogenizers. The homogenate was centrifuged to pellet debris and separate out nuclei. Nuclei were then resuspended in a lysis buffer and incubated at 50C for ∼4 hours, mixing the tubes by gentle inversions every 45 minutes. The DNA was then purified from the lysate using a standard phenol-chloroform extraction. The lysate was mixed with an equal volume of phenol-chloroform-isoamyl alcohol in phase-lock gel tubes, centrifuged for eight minutes, and inverted for eight minutes on a rocker. An equal volume of chloroform was then added to the phase-lock gel tubes, and again the tubes were centrifuged and inverted for eight minutes each. We precipitated the DNA out of solution by adding 10% volume of 3M NaOAc and 2x volume of cold, 100% ethanol to each tube and gently rocking and inverting the tubes to mix. A wide-bore pipette tip was used to transfer the precipitated DNA to new tubes where it was subsequently washed twice with 70% ethanol. The DNA was pelleted and allowed to air dry for a couple of minutes before being resuspended in 60ul of 1 x Tris-EDTA buffer at 50C for one hour and then for a couple of days at 4C. If the DNA was not fully resuspended after a few days, the DNA was sheared by slowly pipetting twice with a P1000 tip and then allowed a few more days to resuspend. DNA concentration was quantified with Qubit, and Nanodrop absorption ratios were checked to ensure that 260/280 was greater than 1.8 and 260/230 was greater than 2.0.

We prepared the nanopore library following the ONT Ligation Sequencing Kit (SQK-LSK114) protocol, with a couple major modifications. First, we started with ∼4.5 ug of DNA as opposed to the recommended 1ug of DNA. Second, we used the Circulomics Short Read Eliminator (SRE) buffer before starting the official Sequencing Kit protocol to remove the shortest DNA fragments. We then performed the Sequencing Kit’s DNA Repair and End-prep steps using half-reactions to save reagents, without loss of quality in the final product. We also performed the adapter ligation and clean-up steps using half-reactions. We additionally let the adapter ligation reaction incubate for ∼30 minutes instead of the prescribed 10 minutes. Finally, we performed a second round of short-read elimination using the Circulomics SRE buffer. Critically, we washed the DNA with SFB or LFB (interchangeable) from the Sequencing Kit because ethanol denatures the nanopore motor proteins. We loaded ∼350 ng of prepared library onto R10.4.1 flow cells and sequenced on an ONT PromethION 2 following ONT’s protocol with live basecalling set to fast mode. For further discussion and justification of these protocols, please refer to our previous work (Kim et al. 2021, 2024).

### Short-read sequencing

To short-read sequence each of the inbred lines, we used the same DNA extracted for the long-read library preps. ∼10ng of DNA were prepared with the Illumina DNA library preparation kit. We followed the standard protocol, but we used one-fifth reactions to save reagents at no cost to the final quality. All libraries were amplified with 6 PCR cycles. The prepared libraries were then cleaned with beads and pooled by concentration. We checked the fragment size distributions with BioAnalyzer, and the concentration of the library pools were quantified via qPCR by Admera Health. Sequencing was performed on the NovaSeq X Plus by Admera Health to 30x–60x coverage. One inbred line (dmel19) was sequenced a second time to reach the desired coverage. We used BBtools (Bushnell 2014) for adapter trimming the short-read data.

### Basecalling and read-length distribution generation

We long-read sequenced the eight inbred lines to extremely high depths (80–365x; master pools) so that we would have high-enough coverage of ultra-long reads. We basecalled our reads using Dorado (model: “dna_r10.4.1_e8.2_400bps_sup@v5.0.0”) and filtered out reads with a quality score less than 10. To generate our different read-length distribution pools for each inbred line, we downsampled the total pools of reads in various ways. We used the program seqtk (https://github.com/lh3/seqtk) for downsampling. To generate the standard pool of reads, we used “seqtk sample” to uniformly and randomly downsample the master pool to 30times-coverage. To generate the ultra-long set of reads, we used “seqtk seq” with the “-L” argument to set the minimum read length such that we only kept the longest reads that resulted in 30×-coverage. For the minimum-10 kb pools, we first used “seqtk seq” with the “-L” argument to remove all reads less than 10 kb from the total pools. Then we used “seqtk sample” to uniformly and randomly downsample the remaining reads to 30×-coverage. For the lower-coverage ultra-long distributions, we uniformly downsampled the ultra-long read pools to 20×-coverage and 10×-coverage. We used the program seqkit to find the summary statistics of each of our downsampled read pools.

### Resequencing for proper standard-length long reads

There were three lines (dmel12, dmel19, dmel21) whose sequencing runs generated much higher proportions of long reads. As a result, the random, uniform downsampling used to create the standard read-length distributions consistently generated pools with read N50s 2-3x higher for these lines. As consistent read-lengths were paramount to our study, we elected to resequence these three lines using the standard long-read protocol from Kim et al. (2021). All other read-length distributions for these three lines were computationally generated from the master pools like the other lines.

### Long-read assembly pipeline

We followed the haploid assembly pipeline described by Kim et al. (2024). We fully assembled genomes for each of the long-read distributions of each inbred line. Briefly, basecalled reads were assembled with Flye (Kolmogorov et al. 2019) using the “–nano-hq –read-error 0.03” parameters. We removed haplotigs from the contigs using purge_dups (Guan et al. 2020), and we then polished with Medaka for one round. Contaminant sequences and adapters were found and removed using the NCBI Foreign Contamination Screen (Astashyn et al. 2024). We scaffolded the cleaned contigs against the *D. melanogaster* reference assembly (v6.58) using RagTag (Alonge et al. 2022). Repetitive sequences were found and soft-masked with RepeatModeler (Smit and Hubley 2008) and RepeatMasker (Smit et al. 2013). Assembly metrics were found using the command “gt stat” from the program GenomeTools (Gremme et al. 2013). We determined BUSCO completeness with the program compleasm (Huang and Li 2023), using the “diptera_odb10” lineage. We used Yak (Cheng et al. 2021) to calculate the QV for all of our assemblies. We generated the k-mer database using our short-read data for each line. We reported the “adjusted” QV value, which is more stable with variable sequencing coverage.

### Long-read structural variant calling

All long-read read pools and assemblies were aligned to the *D. melanogaster* reference assembly (v6.58) using minimap2 (Li 2018). Reads were mapped with the parameter “-x map-ont” and assemblies were mapped with the parameter “-x asm5”. We used minimap2 as it has been specifically refined for R10.4.1 Nanopore reads, and it is recommended by Oxford Nanopore. Previous work did not find significant improvements in variant calling when using a different aligner over minimap2 (Liu et al. 2024). We chose five well-known, structural variant callers that used a variety of algorithms. Structural variants were identified for each read-length distribution in each strain with sniffles2 (Smolka et al. 2024), cuteSV (Jiang et al. 2020), DeBreak (Chen et al. 2023), svim-asm (Heller and Vingron 2021), and PAV (Ebert et al. 2021). We required a minimum read-mapping quality of 20 and a minimum variant length of 50 bp for all callers except PAV, which has its own read-mapping quality thresholds.

For each read-length distribution, structural variants were called in each inbred strain by all five callers, resulting in five independent variant calling format (VCF) files per strain. We used bcftools to filter each of the five VCF files to only keep variants with the “FILTER = PASS” and “PRECISE = 1” flags. We additionally filtered out all variants that did not overlap the euchromatic regions as defined in Chakraborty et al. (2018) (Supplementary Table 2). We only kept insertions, deletions, duplications, and inversions. We then used a custom script to recode duplications as insertions in the VCF files. This was done to allow for proper merging, as duplications were identified as duplications by some callers and insertions by others. We merged the five VCF files for one strain into a single VCF file using Jasmine (Kirsche et al. 2023), and we filtered out all structural variants that were not called by at least two independent callers (Jasmine parameter “min_support = 2”). Finally, for each read-length distribution we merged the eight strain VCF files into a single population VCF file using Jasmine.

### Short-read structural variant calling

Paired reads were first aligned to the *D. melanogaster* reference assembly (v6.58) with bwa-mem (Li 2013) via the “fq2bam” command within Parabricks (https://docs.nvidia.com/clara/parabricks/latest/index.html). Resulting bam files were merged with sambamba (Tarasov et al. 2015). Structural variants were called from short-read data using Manta (Chen et al. 2016), Delly2 (Rausch et al. 2012), Lumpy (Layer et al. 2014), and GRIDSS2 (Cameron et al. 2017). Variant callers were primarily used with default parameters. Manta’s break-end calls were converted to inversions with the “convertInversion.py” command. GRIDSS2 calls all variants as break-ends, so the included script “gridssToBEDPE” was used to convert the calls to insertions, deletions, duplications, and inversions. Short-read structural variant calls were subjected to the same filtering steps as the long-read calls. Variants were required to be called by at least two callers when merging at the strain-level. Jasmine was used for all merging steps.

### Manual validation of structural variants

To verify the presence or absence of called structural variants in each of the inbred lines, we used Jbrowse2 (Diesh et al. 2023) to visualize the eight read alignments from the ultra-long read-length distribution. We elected not to visualize the master read pools as the extremely high coverages made loading all eight strains into visualization programs intractable. We required that the read alignments be in concordance with the VCF entries for multiple criteria for a structural variant to be positively verified. The read alignments had to show the same variant type, genomic location, and presence and absence across the eight lines as described in the VCF entry. When variants, specifically insertions, had small size differences between strains, we used UCSC’s Genome Browser (Perez et al. 2025) and the BLAT tool (Kent 2002) to ensure that all variants contained the same sequence. To maintain consistency, a single person performed all of the manual validation.

While inbred lines are generally treated as completely homozygous, there are still regions of heterozygosity (Frankham et al. 1993; Rumball et al. 1994; Powell and Evans 2017; De Kort et al. 2022) and we observed structural variants that were clearly heterozygous. We were able to differentiate heterozygous variants, even at low read frequencies, from sequencing errors or false alignment signatures. Heterozygous variants are often found in distinct haplotypes with different SNPs and indels in the surrounding genomic regions. All reads containing true heterozygous variants will similarly contain the same heterozygous SNPs and indels.

We briefly describe key alignment details for each type of structural variant. Non-tandem-duplication insertions should be found at the same breakpoint with the same length across the reads (Supplementary Fig. 1 in File S1). Tandem-duplication insertions should have the same length, but will not necessarily be found at the same breakpoint (Supplementary Fig. 2 in File S1). In shorter reads, duplications will be detected due to significant increases in coverage (Supplementary Fig. 3 in File S1). Depending on what the aligner deemed as the “duplicated” sequence, the breakpoint can range from the start of the first copy to the start of the second copy. Deletions should have the same length and same start and end breakpoints across reads (Supplementary Fig. 4 in File S1). Both insertions and deletions have specific graphical representations in JBrowse2, but in certain cases where the insertion or deletion is sufficiently large, it can be represented as a gap in aligned sequence. Inversions should have the same length and the same start and end breakpoints across reads. Small inversions that can be captured by single reads should clearly show the inverted sequence relative to the reference (Supplementary Fig. 5 in File S1). Large inversions will be characterized by split-mapped reads (Supplementary Fig. 6 in File S1). The reads on one side of a breakpoint should appear to map far away (in reference coordinate space) to the other end of the inverted sequence.

In the event of a false positive structural variant call, we classified the error into one of five broad categories. If the error stemmed from read misalignments of transposable or mobile elements or other repetitive elements as defined by RepeatMasker, then we classified the error as a “repetitive element” error (Supplementary Fig. 7 in File S1). If the variant error was caused by misalignments in a repetitive genomic locus (of non-repetitive-element sequence) or in a locus with intersecting or overlapping structural variants, the error was considered a “complex region” error (Supplementary Fig. 3 in File S1). If a variant call was found to be the summation of two smaller structural variants in the alignment, the smaller variants had differing sequence, and the region was not repetitive, then the call was considered a “strain merging” error (Supplementary Fig. 8 in File S1). If a putative variant call was found across multiple strains, and the variants in different strains were found to actually be different structural variants, then the putative call was assigned as a “population merging” error (Supplementary Fig. 2 in File S1). If a putative variant call did not correctly include a strain that actually had the variant, then the VCF was checked to see if the call was made at the strain level and incorrectly merged at the population level. If that was the case, then it was also designated as a population merging error. If the variant call did not exist at the strain level, then one of the other error designations was used. Finally, if a call did not fall into any of these categories, it was binned into “other” errors.

## Results

### A ladder of read-length distributions

**Fig. 2.**
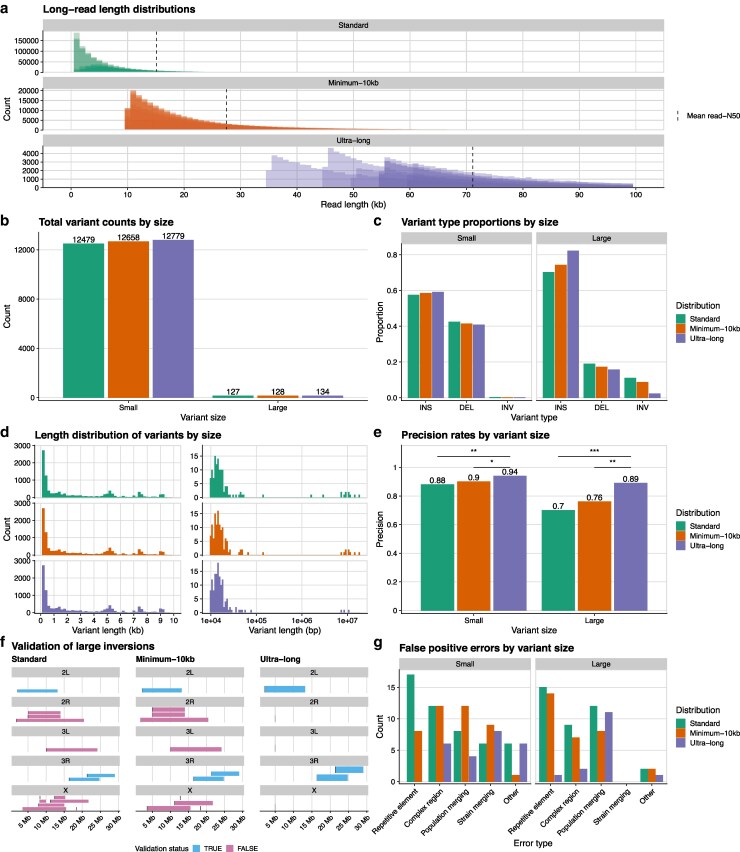
Ultra-long data finds the most comprehensive set of structural variants. a) Read-length histograms of the three, main long-read-length distributions used in this study. Each histogram shows the individual read-length distributions for each of the eight inbred lines. The dashed line represents the mean read N50 of the eight lines for that distribution. For clarity, the histogram only shows reads up to 100 kb, however read lengths extend up to 1 Mb at minimal frequency. b) The total structural variant counts found by each read-length distribution, broken down by variant size. Small variants were defined as being less than 10 kb in length, while large variants were equal to or longer than 10 kb. c) The relative proportions of variant types found by each read-length distribution, broken down by variant size. d) The variant length histograms for small (left) and large (right) variants found by each read-length distribution. e) The precision rate of each read-length distribution as determined by manual validation, broken down by variant size. f) Chromosomal maps of all large inversions found by each read-length distribution, colored by whether those inversions are real, as determined by manual validation. g) The false positive errors as determined by manual validation for each read-length distribution, broken down by variant size. Repetitive element errors stemmed from incorrect alignment of mobile or repeat elements. Complex region errors were caused by incorrect alignments in highly repetitive regions or in regions with intersecting structural variants. Population merging errors were caused by incorrect genotyping of specific variants across the eight strains. Strain merging errors were when very small indels within a strain were incorrectly merged together and called as a larger variant. All other errors were binned into the “Other” category. * = *P* < .05; ** = *P* = .001; *** = *P* < .0001.

To investigate the effects of long-read length on automated structural variant calling, we performed ONT long-read sequencing on eight inbred *D. melanogaster* lines to generate “master” read pools of ∼80-365x coverage. We then computationally downsampled these reads to generate 30×-coverage pools of different read lengths (Fig. 2a). 30×-coverage has been a standard value for variant calling and genome assembly (Bentley et al. 2008), and the assembler we used was extensively tested with 30×-coverage genomic data (Kolmogorov et al. 2019). In *D. melanogaster*, previous work has shown that Phred-scaled consensus accuracy greatly improves up to 30× coverage. Quality more gradually improves past 30× coverage with lesser returns (Kim et al. 2024). We quantified the read-length distributions using the read N50 statistic, which represents the minimum length of reads such that 50% of the total data are represented in reads that length or longer. We uniformly downsampled the master pools of each line to generate a “standard” read-length distribution, resulting in read N50s ranging from 10 kb–19 kb (mean=15 kb; Table 1; Supplementary Table 3). This read N50 range was in line with previous *D. melanogaster* structural variant studies (Chakraborty et al. 2018, 2019; Rech et al. 2022). In this study, we followed the ONT documentation and defined the “ultra-long” distributions as having read N50s greater than 50 kb. Our ultra-long distributions were more meaningfully defined not by a statistic but by being exclusively composed of reads that are long enough to span challenging regions of the genome. To generate the ultra-long read-length distributions (read N50s 57 kb–87 kb; mean=71kb; Table 1; Supplementary Table 3), we enforced a minimum read-length cutoff (35 kb–60 kb; mean=52 kb) such that we had 30×-coverage of the longest reads from the master read pools. We finally generated a “minimum-10 kb” read-length set (read N50s 22 kb–38 kb; mean = 27 kb; Table 1; Supplementary Table 3), where all reads less than 10 kb were discarded from the master read pools before we performed uniform downsampling to 30×-coverage. The minimum-10 kb read pools were designed to mirror the standard read pools but without the shortest reads. This was to control for the fact that the ultra-long read pools had a minimum length threshold, and the presence of shorter reads could create alignment errors that affected structural variant calling more significantly than the gains from increased read lengths. We chose 10 kb as the threshold in order to remove all reads that could not span the largest transposable elements, which are 10 kb in length in *D. melanogaster*.

**Table 1.** Sequencing and assembly statistics for each of the read-length distributions averaged across the eight inbred lines.

Distribution	Coverage (145Mb)	Min. read length (bp)	Read N50 (bp)	Contig N50 (Mb)	Total scaffold length (Mb)	Scaffold N50 (Mb)	Total scaffolds	Busco score	QV
Master	237.8	–	–	–	–	–	–	–	–
Standard	31.6	5	15,085	13.6	129.6	23.8	43.5	99.5	39.88
Minimum-10 kb	29.7	10,000	27,448	14.6	132.3	24.4	19.5	99.5	39.93
Ultra-long	30.3	51,875	71,184	17.8	137.1	25.1	13.5	99.5	39.26
Ultra-long-20×	20.2	51,875	71,206	19.3	134.7	24.7	14.8	99.5	39.56
Ultra-long-10×	9.9	51,875	71,254	9.8	133.6	24.7	15.1	99.3	36.40
Short-read	46.6	10	150	–	–	–	–	–	–

Note that the master read pools and the short-read pools were not assembled. For line-specific statistics, please see Supplementary Table 3.

### High-quality genome assemblies from long-read data

To make use of assembly-based structural variant callers, we assembled each of the standard, ultra-long, and minimum-10 kb read-length pools into full genomes. As we sequenced established inbred lines, we did not use haplotype-aware assembly methods to generate phased genomes. Overall, these assemblies were highly contiguous (Table 1; Supplementary Table 3), with the standard assemblies having contig-N50s averaging 13.6 Mb in length. The ultra-long reads generated near-chromosome-level assemblies, with contig-N50s averaging 17.8 Mb. The minimum-10 kb assemblies fell in the middle with contig-N50s averaging 14.6 in length. High contiguity, especially in euchromatic regions, resulted in very high degrees of benchmarking universal single-copy ortholog (BUSCO) completeness (all > 99%). Across the read-length distributions, our assemblies had high base-level accuracy, with average QV values between 36 and 40.

### Ultra-long reads and assemblies greatly increase precision for structural variant calling

To call structural variants from long-read data, we employed five, well-known, automated structural variant callers. Sniffles2 (Smolka et al. 2024) and cuteSV (Jiang et al. 2020) use strictly read-based alignment approaches, DeBreak (Chen et al. 2023) uses a read-based approach coupled with local reassembly around the putative structural variant, and svim-asm (Heller and Vingron 2021) and PAV (Ebert et al. 2021) utilize assembly-based alignment approaches. For each read-length distribution, we aligned the 30×-coverage read pools and the resulting assemblies to the *D. melanogaster* reference genome (v6.58). We limited our analyses to insertions, deletions, and inversions, as these variant types were universally recognized by our variant callers. Most of the callers we used also had the ability to detect duplications, and we converted those calls to insertions so that they could be properly merged.

All of our alignments and structural variant calls were subject to multiple filtering steps, including minimum alignment scores, minimum and maximum genome coverage, and “PASS” and “PRECISE” variant flags (see Materials and Methods). Leveraging a strategy employed by previous studies to reduce the false positive rate, we required all variants to be independently found by at least two automated callers (Li et al. 2023; Mérot et al. 2023; Couper et al. 2025; Jensen et al. 2025). In line with other *D. melanogaster* structural variant studies (Chakraborty et al. 2018, 2019; Rech et al. 2022), we chose to only look at structural variants found in the euchromatic regions of the genome (Supplementary Table 2). We chose conservative filtering steps throughout our variant calling pipeline as false positive calls at the strain level could lead to significant artifacts at the population level when variant calls were merged.

Since few validated structural variation datasets currently exist for *D. melanogaster* (Chakraborty et al. 2018, 2019), and none existed for our specific set of inbred strains, we validated structural variant calls using a manual approach (Supplementary Table 5). We visualized the ultra-long read alignments of each strain at the putative variant locus with Jbrowse2 (Diesh et al. 2023). If manual validation found a variant to be present in exactly the same inbred lines as the automated callers, then the variant was designated as a true positive. Without ground-truth variant calls, our analyses were necessarily blind to false negatives, so we strictly calculated the precision of the structural variant callers. We divided our manual validation into two categories, looking at the performances of the callers on “small” structural variants, which we defined as variants with a length less than 10 kb, and on “large” structural variants greater than or equal to 10 kb. We chose this threshold as it was just smaller than the read N50s of our shortest long-read distribution and because the largest transposable elements in *D. melanogaster* are just under 10 kb in length.

When looking at small structural variants, we found subtle differences in the types of variants detected by each of the three read-length distributions. The standard data found 300 (2%) fewer structural variants than the ultra-long data (Fig. 2b; Supplementary Table 4). We next checked the proportions of our variant types within these counts, and we found that the ultra-long data called the largest proportion of insertions and the smallest proportion of deletions, while the standard data showed the inverse (Fig. 2c). Though the small inversion proportions were less than one percent for all read-lengths, the standard data found the most (19) while the ultra-long data found the fewest (14). Finally, the variant length histograms showed no qualitative differences between the datasets (Fig. 2d). Each histogram showed that most structural variants were less than 1,000 bp. There were additional modes representing variation caused by different mobile element families (Mérel et al. 2020), a pattern that has been observed in other species (Weissensteiner et al. 2020; Ebert et al. 2021; Li et al. 2023).

Despite the subtle differences in the makeup of variants called by the different distributions, the small-variant precision rates for the standard and ultra-long read-length distributions differed by nearly 10% (Fig. 2e). We randomly sampled and validated 400 small variants from each of the callsets. Of the 400 standard, small structural variants, 350 (87.5%) were true positive calls. The ultra-long data found a statistically significant increase in true positive calls (chi-square test; p=.001 against standard), with 376 (94%) of the 400 small variants being true positives. While the standard read-length distribution accurately called many structural variants less than 10 kb in length, the ultra-long data ultimately captured more small variants and with higher precision.

We repeated our analyses, next focusing on large structural variants with lengths of at least 10 kb. Again, each of the read-length distributions recovered very similar numbers of total large variants, with the standard data finding just seven fewer (5%) than the ultra-long data (Fig. 2b). The proportion of insertions differed, with 110 (82%) of the ultra-long variants being insertions, while the standard data found only 89 (70%) insertions (Fig. 2c). The standard large variants included 14 (11%) inversions, while the ultra-long data found just three (2%) inversions. The standard data was also composed of more deletions (24; 19%) than the ultra-long data (21; 16%). The large-variant length histograms showed almost all variants found by the ultra-long data were in the 10–100 kb range, while the standard variants had a second mode around 10 Mb (Fig. 2d).

Ultimately, the large-variant precision rate was much higher for the ultra-long data than for the standard dataset. We manually validated all of the large structural variant calls from the standard and ultra-long data. 121/134 (90%) of the ultra-long large variants were found to be real (Fig. 2e). Strikingly, the large variant calls from the standard data were much less accurate (chi-square test; P<.0001) as only 89/127 (70%) were true positives. Again, the ultra-long data returned the largest and most accurate set of structural variants. We specifically compared the sets of large structural variants called by the two datasets and found that about one quarter of variants were unique to the ultra-long data (35/134) and to the standard data (30/127). 30 of the 35 (86%) of the unique ultra-long variants were validated as real, while just 5 of the 30 (17%) unique standard variants were true positives. We found that the true positive calls unique to the standard dataset occurred in genomic regions where the ultra-long data had abnormally low coverage.

While the ultra-long data contained reads that were on average ∼7x longer than the standard reads, the ultra-long data also contained no reads less than 35 kb. It was possible that the improvements seen with the ultra-long data came from the lack of small reads, which would often align poorly, rather than an excess of longer reads. We repeated our analyses with the minimum-10 kb data, expecting the minimum-10 kb data to show similar results to the ultra-long data if indeed that was the case. We found that the minimum-10 kb results always fell between the standard and ultra-long values (Fig. 2). Ultimately, the minimum-10 kb precision rates for both small (358/400; 89.5%) and large variants (97/128; 76%) were significantly lower than the ultra-long precision rates (chi-square test; P<.05 for small; P=.001 for large), suggesting that the removal of the shortest reads was not the most significant factor to the ultra-long data’s high precision. Rather, the length of the reads in the ultra-long dataset was crucial to correctly aligning and assembling problematic regions with structural variants (Nurk et al. 2022).

Chromosomal inversions are well-characterized in *D. melanogaster* as being adaptive variants (Kapun and Flatt 2019; Kapun et al. 2023; Nunez et al. 2024), thus it is of particular importance to be able to call them correctly. Both the standard (14) and minimum-10 kb (11) called significantly more large inversions than the ultra-long data (3) found. Strikingly, all three of the inversions found in the ultra-long data were known cosmopolitan inversions, and our manual validation confirmed their presence in our inbred lines. The cosmopolitan inversions happened to be heterozygous in these lines, and our reference-guided scaffolding assembled the standard orientation. Despite this, we were still able to plainly detect the inversions using read-based methods, highlighting the benefits of including diverse variant calling algorithms. While the other two read-length distributions also found these three cosmopolitan inversions, none of the remaining inversion calls were real (Fig. 2f).

### The majority of structural variants are found by both read-based and assembly-based callers

We used both assembly-based and read-alignment-based callers to find structural variants. Distinct challenges in genome assembly and read-to-reference alignment can lead to callers finding different sets of variants, and significant differences in those calls could confound our read-length analyses with alignment or assembly quality issues. For each of our read-length distribution datasets, we tracked whether each individual variant (a single variant call in a single sample) was found by just assembly-based callers, by just read-based callers, or by both types of callers. We found that the proportions of variants in each of these categories did not change with read length (Supplementary Table 6). The vast majority (84–85%) of our structural variants were found by both types of callers. The next 13–14% of structural variants were found exclusively by read-based callers. The final 1–2% of variants were found exclusively with assembly-based callers.

We next asked if the same detection methods were used to find non-singleton variants across samples. Again, we observed that these proportions were invariant to read length (Supplementary Table 7). We found that ∼95% of non-singleton variants were detected by read-based and assembly-based methods. ∼4% of non-singleton variants were found using exclusively read-based methods, and ∼1% of non-singleton variants were found using exclusively assembly-based methods. The drop in the proportion of variants that were found using exclusively read-based methods is attributed to the fact that many of these variants were found by both methods in a different sample and merged together.

We reassessed the variants we manually validated in the context of the detection methods. We observed that variants called by both types of detection methods had very high precision (86–96%) for all read lengths and variant sizes (Supplementary Fig. 9). Small ultra-long variants found by both detection methods had a statistically significant increase in precision compared to small variants found by both detection methods in the standard dataset. Variants that were found by only one detection method had a much wider range of precision values (20–92%) (Supplementary Fig. 9). The precision values of these variants scaled with read length, with the ultra-long variants having the highest precision, nearly on par with ultra-long variants called by both detection methods. It should be noted that for certain categories (such as large variants found only with assembly-based methods) there were very few variants, potentially leading to poor estimations of precision (Supplementary Table 8).

### Misalignments of transposable elements and complex repetitive regions primarily cause false positives in long reads

To better understand why automated structural variant callers make errors, we categorized the 199 false variant calls (both small and large) from our three long-read datasets into five categories (Fig. 2g). In more than half of the false positives, reads containing mobile or repeat elements (“repetitive element” errors) or reads in complex regions were incorrectly aligned, leading to false structural variant alignment signatures. Complex regions were defined as loci with non-repeat-element repetitive sequence. A number of complex region errors came from already known, problematic loci like the Cyp6g1/2 locus (Schmidt et al. 2010) (Supplementary Fig. 3 in File S1), the Stellate gene cluster (Tulin et al. 1997), and exon 16 of Ank2 (Chakraborty et al. 2019). These types of errors stemmed from having reads too short to fully span both the repetitive or complex loci and their surrounding genomic regions. As a result, the aligner incorrectly mapped or split-mapped reads. Repetitive element errors led to all false, large inversion calls. At each of the fake breakpoints of the false inversions, there were either transposable element insertions or deletions. Reads that could not span the full transposable element event at one “breakpoint” were incorrectly split-mapped to the event at the other “breakpoint”, creating a fake alignment signature that looked like an inversion to the variant callers (Supplementary Fig. 7 in File S1). The ultra-long dataset accounted for just 8% (9/106) of false positives stemming from repetitive elements or complex regions, with eight of those nine calls being complex region errors. The single ultra-long “repetitive element” error was from an incorrectly mapped sequence ∼80 kb in length that was almost exclusively transposable elements (Supplementary Fig. 10 in File S1).

The secondary source of false positive variant calls was merging errors, either at the population level or strain level. “Population merging” errors occurred when dissimilar variants at the same genomic locus across different strains were merged and considered the same variant across those strains (Supplementary Fig. 2 in File S1). 31 of the 55 “population merging” errors occurred in large variants, and 73% (11/15) of the ultra-long large false positives were due to this type of error. A number of these cases involved very large structural variants (>20 kb) in regions with variable read coverage across the strains, leading to these variants being correctly called in only a subset of the strains. “Strain merging” errors occurred when very small indels (often <50 bp) in one strain were incorrectly grouped together to form a single, larger variant call (Supplementary Fig. 8 in File S1). Automated callers will often left-align or right-align variants in repetitive regions, however in non-repetitive regions this alignment shift can incorrectly merge small variants together. We found all 23 of the “strain merging” errors when validating “small variants”. None of the read-length distributions were significantly biased for or against either of the types of merging errors.

We binned the rest of the false positive error calls into “other” errors, which constituted less than 10% of all false positive calls. It was much more difficult to determine the reason for these errors, and in certain cases we could only guess at the cause. These false positives could have been due to errors from sequencing or assembly. For example, we observed a large, inverted duplication call perfectly captured in a single read. In reality, this could have been due to the “unzipped”, second strand of the DNA molecule being immediately sequenced in the same nanopore after the first strand and then incorrectly counted as a single molecule of DNA. The errors also could have come from stochastic bumps or dips in read coverage, either due to downsampling or uneven sequencing. We observed instances of multiple, unclipped reads starting or ending at the same locus, leading to a rapid change in sequencing coverage that triggered a variant call. In a limited number of cases (< 2%), we could not determine the cause of the false positive variant call.

### Long-read structural variant calling loses precision at lower coverage

Generating ultra-long reads from *D. melanogaster* is a relatively time-consuming and expensive process as compared to other long-read protocols. To see if we could make ultra-long reads more accessible, we uniformly downsampled our ultra-long read pools from 30×-coverage to 20×- (ultra-long-20×) and 10×-coverage (ultra-long-10×) and then assembled genomes for each of the eight inbred lines (Table 1; Supplementary Table 3). We then called structural variants from the ultra-long-20× and ultra-long-10× data using the same pipeline and compared them to our original ultra-long variant calls.

**Fig. 3.**
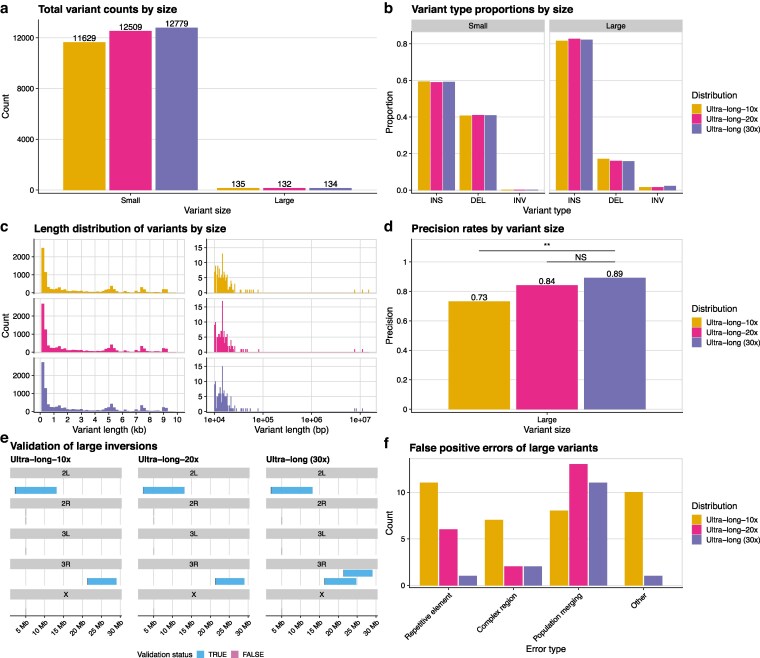
Ultra-long data loses variant-calling precision as coverage decreases. a) The total structural variant counts found by each ultra-long-read coverage distribution, broken down by variant size. b) The relative proportions of variant types found by each coverage distribution, broken down by variant size. Small variants were defined as being less than 10 kb in length, while large variants were equal to or longer than 10 kb. c) The variant length histograms for small (left) and large (right) variants found by each coverage distribution. d) The precision rate for large structural variants of each coverage distribution as determined by manual validation. e) Chromosomal maps of all large inversions found by each ultra-long-read coverage distribution, colored by whether those inversions are real, as determined by manual validation. f) The false positive errors of large variants as determined by manual validation for each ultra-long coverage distribution. Repetitive element errors stemmed from incorrect alignment of mobile or repeat elements. Complex region errors were caused by incorrect alignments in highly repetitive regions or in regions with intersecting structural variants. Population merging errors were caused by incorrect genotyping of specific variants across the eight strains. Strain merging errors were when very small indels within a strain were incorrectly merged together and called as a larger variant. All other errors were binned into the “Other” category. NS=Not Significant; ** = *P* = .001.

The 20×-coverage data called just a couple hundred (2%) fewer total variants than the 30×-coverage ultra-long data, while the 10×-coverage data called almost 10% (1,150) fewer structural variants (Fig. 3a). Despite the spread in total variants called, all three ultra-long distributions had very similar proportions of variant types and showed very similar variant length distributions (Fig. 3b). Each of the different coverages successfully recovered the secondary modes corresponding to different mobile element families. Like the ultra-long dataset, both the 20×-coverage and 10×-coverage datasets called just a handful of structural variants larger than 1 Mb (Fig. 3c). In spite of these broad similarities, the lower-coverage datasets did not match the precision of the 30×-coverage ultra-long dataset. We manually inspected all large variant calls for the ultra-long-20× and ultra-long-10× datasets and found that the ultra-long-20× variant set had an 82% precision rate, while the ultra-long-10× set had just a 72% precision rate, which were both lower than the 30×-coverage dataset’s precision of 89% (Fig. 3d). Only the ultra-long-10× data’s precision rate (chi-square test; P=.001) was statistically significantly lower than that of the original 30×-coverage’s rate, however neither of the lower coverage datasets was able to recover all of the large inversions found by the original 30×-coverage data (Fig. 3e).

A substantial fraction of these errors, especially for the 10x-coverage data, came from misalignments from repetitive elements and complex regions (Fig. 3f). The decreases in coverage meant the aligner had to work with fewer reads that spanned specific loci. Additionally, with an average of just 10 reads per genomic locus, the relative frequency of poor alignments was much higher, leading to more errors. We observed an increase in the number of “population merging” errors in the ultra-long-20x data. In these cases, computational downsampling led to stochastic variation in the sequencing coverage of the variant locus across strains, creating population merging errors. Finally, 10 false calls from the ultra-long-10x data were categorized in “other” errors. Many of these were seemingly heterozygous variants that had too low coverage in the 10x-coverage dataset to be accurately called. Even though we were using inbred lines, it is well known that inbred lines can maintain heterozygosity of both SNPs and structural variants (Frankham et al. 1993; Rumball et al. 1994; Powell and Evans 2017; De Kort et al. 2022).

### Short-read data do not accurately capture the full breadth of structural variation

Though the vast majority of existing genomic data is short-read data, it is well-established that long reads outperform short reads in structural variant calling (Chakraborty et al. 2018, 2019; Kosugi and Terao 2024; Pei et al. 2024; Jensen et al. 2025). We additionally tested short-read data in our pipeline to better understand the limitations of short-read-based structural variant calling in *D. melanogaster*. We sequenced each of the eight lines with Illumina to generate standard, 2×150 bp paired-end, short-read data to an average of 46x-coverage (Table 1; Supplementary Table 3). For the short-read data, we called variants with four established, short-read-based callers, Manta (Chen et al. 2016), Delly2 (Rausch et al. 2012), Lumpy (Layer et al. 2014), and GRIDSS2 (Cameron et al. 2017). Again, we merged with Jasmine, and we required all variants to be independently found by at least two of the four automated callers. We compared the resulting structural variant calls to those found by the standard and ultra-long long-read datasets.

**Fig. 4.**
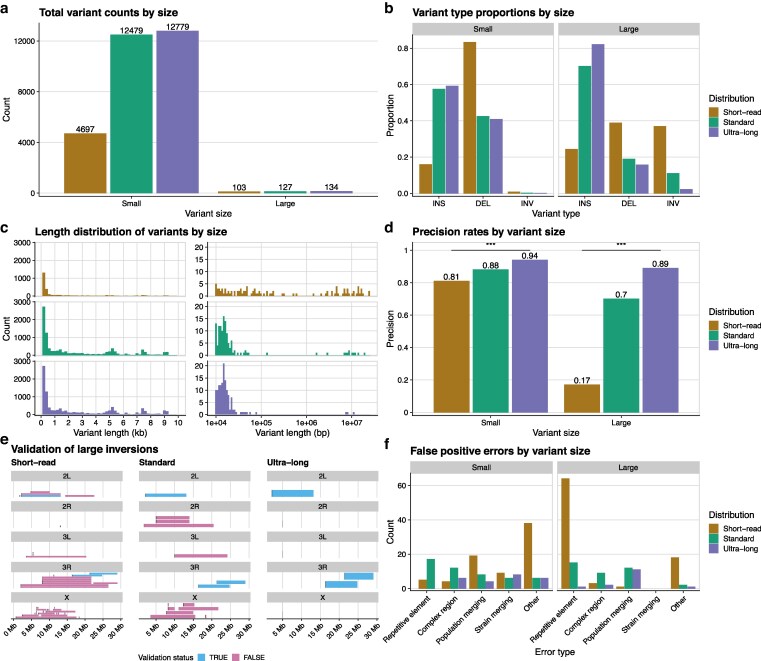
Short-read data recovers a biased set of variants with low precision. a) The total structural variant counts found by each read-length distribution, broken down by variant size. b) The relative proportions of variant types found by each read-length distribution, broken down by variant size. Small variants were defined as being less than 10 kb in length, while large variants were equal to or longer than 10 kb. c) The variant length histograms for small (left) and large (right) variants found by each read-length distribution. d) The precision rate for large structural variants of each read-length distribution as determined by manual validation. e) Chromosomal maps of all large inversions found by each read-length distribution, colored by whether those inversions are real, as determined by manual validation. f) The false positive errors of variants as determined by manual validation for each read-length distribution, broken down by variant size. Repetitive element errors stemmed from incorrect alignment of mobile or repeat elements. Complex region errors were caused by incorrect alignments in highly repetitive regions or in regions with intersecting structural variants. Population merging errors were caused by incorrect genotyping of specific variants across the eight strains. Strain merging errors were when very small indels within a strain were incorrectly merged together and called as a larger variant. All other errors were binned into the “Other” category. *** = *P* < 1e-4.

Unsurprisingly, we found differences between the short-read data and the long-read datasets, as the short-read data called just 4,697 small variants, or 37% of what the long-read datasets found (Fig. 4a). When looking at the proportions of small variant types found, the short-read data had a striking lack of insertions (14%) and a huge percentage of deletions (84%) (Fig. 4b). The length histogram of the short-read variants did not display secondary modes corresponding to mobile element families like the long-read distributions had shown (Fig. 4c). The two previous results taken together show that, as expected, short-read data poorly recovered transposable-element-mediated insertions. The reads were too short to effectively capture the large, multi-kilobase insertions. We manually validated 400 random, small structural variants from the short-read data and found that the precision (81%) was worse than all of the long-read callsets (chi-square test; P<1e-4 against ultra-long data) (Fig. 4d). With both a lower precision rate and a severe deletion bias, structural variant calling with short-read data failed to create an accurate picture of the small structural variant landscape (Chakraborty et al. 2018, 2019).

When comparing the large structural variant calls, we again found that the short-read data severely under-called insertions and over-called deletions and inversions compared to the long-read data, even though the overall numbers were much closer (Fig. 4b). The short-read data notably called the most large inversion (38) out of any read-length distribution. The large-variant size distribution from the short-read data appeared markedly different from any of the long-read distributions, with a much more uniform distribution of variants up to, and past, 10 Mb (Fig. 4c). We manually validated each of the 103 large, short-read variants and found that just 17 (17%; chi square test; P<1e-4 against ultra-long data) were real variants (Fig. 4d). Nearly all (35/38) of the large inversions called by the short-read data were false positives (Fig. 4e). The vast majority of the large false positive calls came from spurious signatures generated by misaligned TE and DNA repeat sequences (Fig. 4f). The short-read variant calls had a higher incidence of “other” errors as compared to the long-read data, especially for small variants. We found these to be largely the same types of errors, as found in the long-read data, just at a higher frequency. There did not appear to be an error type unique to short-read data.

## Discussion

We report significant shifts in automated structural variant calling precision at the population level when systematically varying read length within *D. melanogaster*. Our longest long-read-length distribution, the ultra-long reads (minimum read-length=35 kb; average read N50=71 kb), called more structural variants, and at a higher precision, than any other read-length distribution. We found that the total number of called structural variants, the number of called insertions, and the precision all increased as the read N50 increased. While all long-read datasets called structural variants less than 10 kb in length with at least 88% precision, only the ultra-long dataset maintained similar precision with variants longer than 10 kb.

In this study, we manually validated more than 2,300 putative structural variant calls at more than 18,000 genomic loci across eight inbred strains. Our manual validation not only allowed us to assess the precision of computational variant calls, but also let us determine the causes behind the false positives. In all datasets except for the ultra-long distribution, misalignments from repetitive elements and complex genomic regions caused more than half of all false positive calls. In these cases, the reads were not long enough to both fully span the problematic region and capture enough surrounding genomic context. As a result, the aligner incorrectly mapped repetitive elements, leading to false structural variant signatures for the callers. Without ground-truth structural variants in our inbred lines beyond the cosmopolitan inversions, we were unable to assess false negatives and measure the recall of our pipeline. In all of the datasets, all three cosmopolitan inversions were correctly found, suggesting that, at least for inversions, our false negative rate was low.

In *D. melanogaster*, the longest mobile elements approach 10 kb, and our results suggest that reads significantly longer than the largest repetitive elements are required to accurately map them. Our ultra-long reads, with a minimum read-length of 35 kb, were able to map these elements, suggesting that reads at least 3x longer than the largest repetitive element may be needed to avoid variant-calling errors from these elements in *Drosophila*. Tandem arrangements of mobile elements can cause further issues and will require even longer reads. The only transposable-element-mediated error in our ultra-long dataset was caused by an ∼80 kb stretch of tandem transposable elements. Additionally, only the 30x-coverage of ultra-long reads was able to accurately call the three cosmopolitan inversions. The two low-coverage ultra-long datasets each recovered just two out of the three inversions. We tested a maximum sequencing depth of 30× across each of our read-length distributions. Greater genomic coverage would generically improve precision, however population-scale datasets must consider financial and technical trade-offs between sequencing individuals more deeply and sequencing greater numbers of individuals. This study provides a baseline for standard long-read sequencing coverage.

Since the repeat element landscape is highly variable across taxa, these results further highlight that accurate structural variant calling is a species-specific problem. *D. melanogaster* harbors a relatively small number of mobile elements, however different copies of some transposable elements can have very high sequence similarity, hindering accurate alignment without additional genomic context. In other organisms, long reads that are 3x the length of the largest repetitive elements is a safe baseline, but more in-depth assessments of the repeat landscape and genomic architecture will be needed to find the optimal read lengths that balance accuracy and cost. In line with previous studies (Chakraborty et al. 2018, 2019; Kosugi and Terao 2024; Pei et al. 2024; Jensen et al. 2025), our short-read data failed to capture large structural variants or insertions, especially those mediated by transposable elements. In species like *D. melanogaster* where so much of the variation is driven by transposable elements, care needs to be taken to address these short-read biases. In organisms that have older, more diverged repeat element copies, shorter long reads may be able to distinguish specific repeat copies without the need for as much surrounding genome context. A key insight for determining that longer read lengths were required was that the majority of our false positive structural variant calls stemmed from misalignments. Manual validation of erroneous variant calls in other species will also be essential for finding optimal long-read lengths.

The primary type of error for the ultra-long reads, and a secondary error contributor to all other read-length datasets, was error from merging variants across individuals. In these cases, structural variant calls either were incorrectly assigned to strains that did not contain the variant or were absent from strains that actually did contain the variant. Merging structural variant calls across different individuals is a complex problem, and our results highlight the importance of developing algorithms that include multiple types of evidence for merging, including breakpoints, variant length, and variant sequence content. We observed an increase in merging errors with our moderate coverage dataset (20×). We might expect an increase of these errors with moderate sequencing depth where falling above or below critical thresholds set by the automated callers is more randomly distributed across multiple individuals (as opposed to low coverage data, where individuals would generally fall below thresholds and high coverage data, where individuals would generally fall above thresholds).

In the context of standard Nanopore sequencing and genome assembly, we find that read length matters for SV calling. Standard length Nanopore reads are able to precisely call the majority of structural variants less than 10 kb, however they are significantly less precise with variants longer than 10 kb. Ultra-long Nanopore reads are able to call both large and small structural variants with extremely high precision. We used the recommended Nanopore-specific parameters for our aligner and assemblers. Altering specific aligner or assembler parameters could improve structural variant calling, but it could also introduce new types of errors. Currently, there does not appear to be a significant difference in variant calling between the gold-standard aligner programs (Liu et al. 2024). We hope this work brings attention to the importance of Nanopore read length distributions in regards to structural variant calling.

In our data, we observed that more variants were found from read-based methods than assembly-based methods, which suggests that our read alignments were higher quality than our assembly alignments. The fact that the proportions of variants found by read-based methods or assembly-based methods was invariant to read length suggested that we reached an upper limit for the quality of our genome assemblies. A new haplotype-aware assembler is now available for noisier Nanopore reads, which will especially improve variant calling in non-inbred individuals (Cheng et al. 2025). Additionally, new Nanopore Duplex reads are approaching HiFi in terms of base accuracy while maintaining read lengths >50 kb, potentially combining the best of both technologies (Koren et al. 2024).

We focused exclusively on ONT long reads, however PacBio HiFi reads are a common, alternative long-read data type used for structural variant calling. Read length may become less important as base accuracy improves, as previous work in *D. melanogaster* has shown that assemblies generated from HiFi reads, which have a maximum length of 25 kb, can effectively call structural variants (Chakraborty et al. 2018, 2019). Our results suggest that the extreme length possible with ONT ultra-long reads could outweigh gains in base-pair accuracy from HiFi reads in specific cases. Certain transposable element families have proliferated very recently, inserting identical copies across the genome (Petrov et al. 2011). This makes it very challenging to correctly place individual transposable element copies based on sequence alone. Surrounding genomic context is required to accurately align and assemble these insertions, and ONT long reads are well-suited to span these regions. Future work will be needed to precisely interrogate the differences in structural variant calling between these two sequencing technologies.

We focused on structural variants at least 50 bp in length, however there are many biologically-relevant variants that fall below this threshold. Recent improvements to Nanopore chemistry have shown comparable small-variant-detection results to short-read methodologies (Kolmogorov et al. 2023). Our pipeline is well-suited to detect these smaller variants as well (a single parameter at the start of variant detection controls the threshold). By leveraging long reads, small variants in complex genomic regions can be accurately found.

The ability to accurately call a much fuller breadth of structural variants, without biases for variant length or type, opens up new possibilities to answer long-standing questions in population genomics and genome evolution. For instance, without variant-type biases, we can accurately compare the selective forces acting on different structural variants. We can capture and assemble multiple copies of gene duplications, including those that are still polymorphic, and accurately delineate the accumulation of substitutions in different copies to infer the selective pressures on newly duplicated genes (Kondrashov 2012). While many examples of adaptive structural variation have been found through concerted efforts on specific genomic loci (Chan et al. 2010; Dodge et al. 2024; Xia et al. 2024), there are many regions across the genome, found by GWAS or other genome-wide statistical scans using SNPs, that are implicated in selective sweeps (Garud et al. 2015), allelic series (Schmidt et al. 2010), and rapid and seasonal adaptation (Rudman et al. 2022; Bitter et al. 2024) which could be underpinned by now-detectable, functionally-relevant structural variation. We firmly believe that accurate, population-level calling of structural genomic events will allow the field to make key discoveries that are currently obscured by the almost-exclusive focus on single nucleotide polymorphisms.

## Data Availability

Supplemental information is available through the GSA FigShare portal (https://doi.org/10.6084/m9.figshare.31301737). All long-read and short-read sequencing data generated in this study have been submitted to submitted to the NCBI BioProject database (https://www.ncbi.nlm.nih.gov/bioproject/) under accession number PRJNA1247986. Raw Nanopore signal data (.pod5) will be provided upon email request due to their large file sizes. All genome assemblies, VCF files, and manual validation results have been uploaded to FigShare (assemblies DOI: https://doi.org/10.6084/m9.figshare.31458934; VCFs DOI: https://doi.org/10.6084/m9.figshare.31458985; Manual validation DOI: https://doi.org/10.6084/m9.figshare.31458883). Detailed Snakemake pipelines for genome assembly and structural variant calling and scripts for data analysis are provided on GitHub (https://github.com/jahemker/drosophila_ultralong_sv_calling). A detailed DNA extraction and library prep protocol can be found on protocols.io (dx.doi.org/10.17504/protocols.io.14egn98wql5d/v1).
